# Impact of the gut microbiome on atherosclerosis

**DOI:** 10.1002/mlf2.12110

**Published:** 2024-04-01

**Authors:** Yuqin Mao, Chao Kong, Tongtong Zang, Lingsen You, Li‐Shun Wang, Li Shen, Jun‐Bo Ge

**Affiliations:** ^1^ Department of Cardiology, Shanghai Institute of Cardiovascular Diseases, Zhongshan Hospital Fudan University Shanghai China; ^2^ National Clinical Research Center for Interventional Medicine Shanghai China; ^3^ Center for Traditional Chinese Medicine and Gut Microbiota, Minhang Hospital Fudan University Shanghai China

**Keywords:** atherosclerosis, coronary heart disease, gut microbiome, metabolites, treatment

## Abstract

Atherosclerosis is a chronic inflammatory metabolic disease with a complex pathogenesis. However, the exact details of its pathogenesis are still unclear, which limits effective clinical treatment of atherosclerosis. Recently, multiple studies have demonstrated that the gut microbiota plays a pivotal role in the onset and progression of atherosclerosis. This review discusses possible treatments for atherosclerosis using the gut microbiome as an intervention target and summarizes the role of the gut microbiome and its metabolites in the development of atherosclerosis. New strategies for the treatment of atherosclerosis are needed. This review provides clues for further research on the mechanisms of the relationship between the gut microbiota and atherosclerosis.

## INTRODUCTION

Atherosclerosis is a degenerative vascular disease characterized by deposition of plaque formed by blood components, including lipids and complex sugars. A common cardiovascular disease, atherosclerosis is a leading cause of death and a serious threat to human life and health^1^. Onset and progression of atherosclerosis involve a variety of mechanisms and risk factors, including disorders of lipid metabolism, a local or systemic inflammatory response, oxidative stress, and vascular endothelial dysfunction, as well as traditional factors, including diabetes, hypertension, obesity, and smoking^2^.

**Figure 1:**
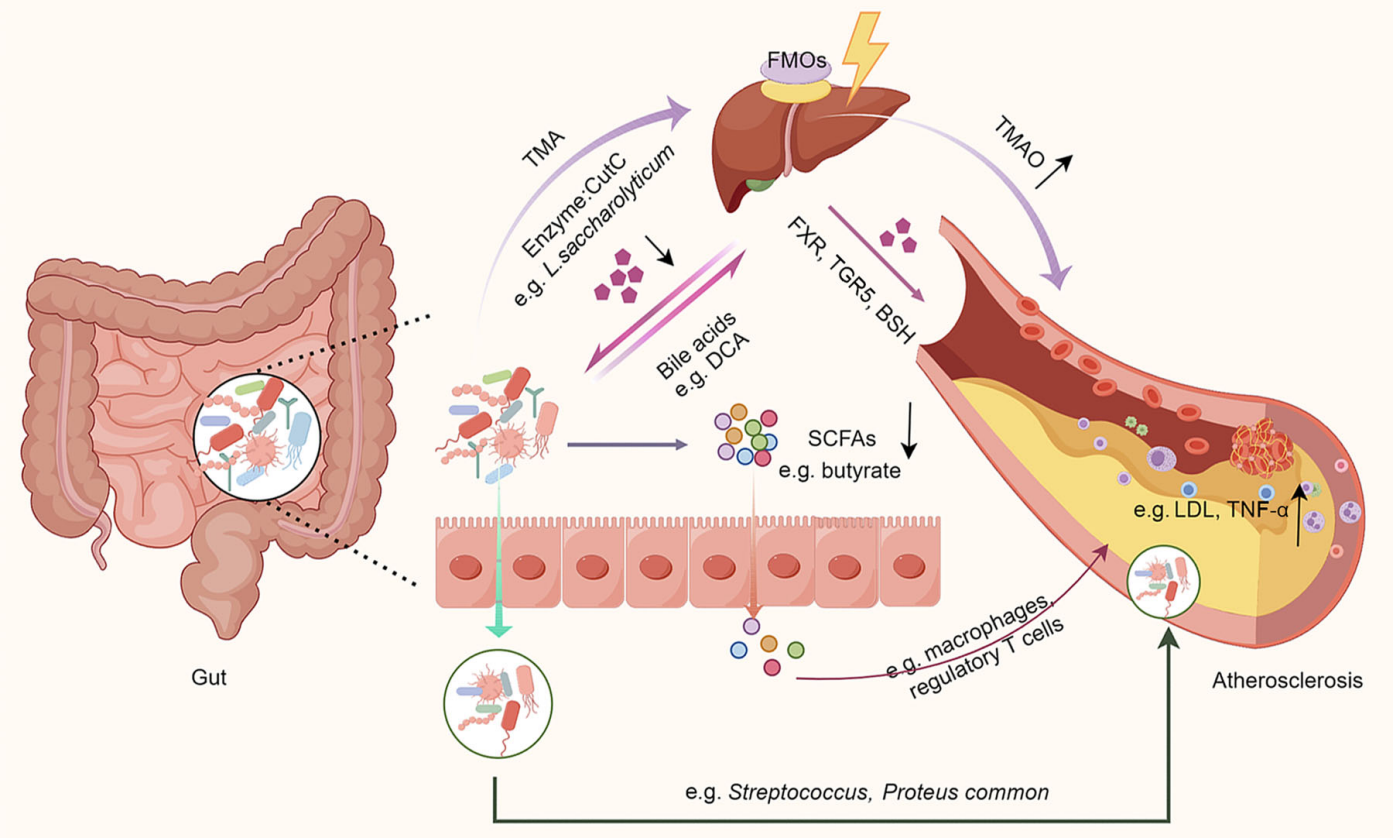
The gut microbiota in atherosclerosis. BSH, bile salt hydrolase; CutC, choline TMA‐lyase; DCA, deoxycholic acid; FMOs, flavin‐containing monooxygenases; FXR, bile acid receptor; *L. saccharolyticum, Lachnoclostridium saccharolyticum*; LDL, low‐density lipoprotein cholesterol; SCFAs, short‐chain fatty acids; TGR5, G protein‐coupled receptor 5; TMA, trimethylamine; TMAO, trimethylamine N‐oxide; TNF‐α, tumor necrosis factor‐α. This figure was drawn by Figdraw (ID:IIYUP00090).

There is substantial evidence to suggest that the gut microbiome may influence the risk factors for atherosclerosis or directly impact atherosclerotic plaque, thereby affecting the onset and progression of atherosclerosis^3^, ^4^ (Figure 1). The microbes in the gut form a complex interacting community of organisms that affect the health of the host^5^. Multiple factors influence the composition, metabolism, and function of host microbes from birth onwards^6^. Many studies have shown that disturbance of the balance of the gut microbiota alters the susceptibility to cardiovascular disease by affecting the immune response, obesity, insulin resistance, atherosclerosis, and susceptibility to thrombosis. Furthermore, microbes in the gut can produce metabolites that communicate with distant organs in the host^7^.

This review discusses the underlying roles and mechanisms of the gut microbiome and its metabolites in the onset and progression of atherosclerosis, and strategies for treatment of atherosclerosis with the gut microbiota as an intervention target.

## GUT MICROBIOME IN THE ONSET AND PROGRESSION OF ATHEROSCLEROSIS

### Gut microbiota in host

The gut microbiota is a complicated community in the host, affecting health and disease through its composition and functionality. The human gut microbiome consists of a wide variety of microbes, including bacteria, fungi, archaea, protozoa, and viruses. The gut microbiota contains about 3 × 10^13^ bacteria, of which most are symbiotic intestinal microbes. The gut microbiota is composed of *Firmicutes, Bacteroidetes, Actinobacteria, Proteobacteria, Fusobacteria*, and *Verrucomicrobia* ^5^. *Bacteroidetes* and *Firmicutes* constitute the majority of gut bacteria, and the *Firmicutes*/*Bacteroidetes* ratio in the gut microbiota is considered to be a critical health indicator^8^. The structure of the gut microbiome varies greatly among individuals and is regulated by host factors and environmental parameters, including lifestyle, diet, and diseases^6^.

Emerging studies have demonstrated that the gut microbiota affects a variety of physiological functions, in particular, metabolism, inflammation, and immunity^6^, ^9^. When the gut microbiota is dysfunctional, some symbiotic species (e.g., *Clostridium difficile* and vancomycin‐resistant *Enterococcus*) grow rapidly and develop pathogenic characteristics, so these symbiotic species are regarded as pathogens. The interaction between the gut microbiota and epithelial cells has multiple critical regulatory functions, including regulation of barrier function, maintenance of homeostasis in the mucosa and host–microbial symbiosis, preventing infection controlling pathological bacterial overgrowth, and regulating metabolism. Therefore, the gut microbiota interacts with immune cells and the mucosal barrier to maintain homeostasis in the intestine.

### Contribution of the gut microbiota to development of atherosclerosis

Atherosclerosis is characterized by vascular cell dysfunction and accumulation of low‐density lipoprotein particles in plaque^10^. Rupture of atherosclerotic plaque can lead to severe atherosclerotic cardiovascular disease, including myocardial infarction and ischemic stroke, which result in considerable morbidity and mortality worldwide. Several types of gut microbes, including *Staphylococcus, Proteus vulgaris, Klebsiella pneumoniae*, and *Streptococcus*, have been found in atherosclerotic lesions and the gut of the same individual, suggesting that the gut microbiome is involved in the onset and progression of atherosclerosis^11^, ^12^.

Patients with atherosclerosis have been found to have higher abundance of opportunistic pathogens and a lower abundance of certain butyrate‐producing bacteria^13^, which suggests that these gut microbes may be involved in atherosclerosis. Moreover, the microbes involved in atherosclerosis vary from population to population. For example, the abundance of *Prevotella* has been found to be relatively low in the Chinese population and higher in the gut among middle‐aged men in eastern Poland^3^, ^14^. *Prevotella* plays a pivotal role in health and disease and its abundance is influenced by factors such as diet, lifestyle, and geographic location^15^. *Prevotella* includes more than 50 different species, and there is considerable genetic diversity among the various strains^16^. Current studies have shown that an increased abundance of *Prevotella* is associated with an increased risk of both local and systemic diseases, including rheumatoid arthritis and low‐grade systemic inflammation^17^, ^18^, ^19^, and the higher abundance of *Prevotella* was suspected to be the mechanism of atherosclerosis in the study performed in eastern Poland. However, the low abundance of *Prevotella* in the Chinese population may be influenced by dietary factors and the pathogenicity of the various strains of *Prevotella*, both of which require further study. Although the formation and stability of plaque can be affected by various types of bacteria, it is not clear which bacterial species play a dominant role in atherosclerosis.

Nielsen et al. found an increased abundance of *Collinsella* in patients with symptomatic atherosclerosis, while shotgun sequencing identified an increased abundance of *Eubacterium* and *Roseburia* in healthy controls^20^. In these patients, changes in the composition of the microbiome were also associated with an increased abundance of genes involved in the inflammatory process. The abundance of gut microbes such as *Bacteroides, Clostridium*, and *Lactobacillus* has been shown to predict coronary artery disease^21^. Compared with controls, the abundance of *Lactobacilli* and the *Firmicutes*/*Bacteroidetes* ratio were increased in the stool of patients with atherosclerosis, while that of *Bacteroidetes* was decreased^21^. These findings require confirmation in independent cohorts. Furthermore, whether changes in the microbiota are the cause of coronary artery disease or simply reflect its severity needs clarification.

Recently, Jie et al. conducted the first large‐scale, case–control analysis of the gut microbiome in 218 patients with atherosclerosis and 187 healthy individuals, and found a significant increase in the abundance of *Enterobacteriaceae* and *Streptococcus* genera and a decrease in the abundance of beneficial microbiota (e.g., *Faecalibacterium prausnitzii*) in the stool of patients with atherosclerosis^14^. The abundance of *Streptococcus* was positively associated with blood pressure, while that of *Enterobacteriaceae* was positively related to myocardial indices. On the basis of these findings, they established a model for prediction of the risk of atherosclerosis based on 47 intestinal bacteria found in the Chinese population^14^, ^22^. They collected stool samples from patients with cardiovascular disease and from healthy controls and found that *Vampirovibrio, Ruminococcus*, and *Eisenbergiella* bacteria were associated with mild coronary artery stenosis, stable angina pectoris, and acute myocardial infarction. Unexpectedly, the prediction model based on gut microbiome indicators was more accurate than clinical indicators in the diagnosis of mild coronary artery stenosis and stable angina pectoris. In patients with acute myocardial infarction, the creatine kinase level was positively associated with the abundance of *Sporobacter* and *Eisenbergiella* while hemoglobin showed negative correlation^23^. However, colonization by multiple bacteria rather than a single pathogen could affect the formation and stability of plaque.

Another study found that proinflammatory microbes promoted formation of inflammatory factors and accelerated development of atherosclerosis in a susceptible mouse strain^24^. This effect was associated with a reduction in short‐chain fatty acid (SCFA) levels but did not seem to be associated with the level of trimethylamine N‐oxide (TMAO). Administration of *Bacteroides vulgatus* and *Bacteroides dorei* in a mouse model of atherosclerosis reduced the level of lipopolysaccharide produced by the gut microbiota, thereby reducing the levels of endotoxin in feces and plasma, enhancing formation of tight junctions in the intestinal epithelium, inhibiting intestinal and systemic inflammation, and attenuating atheromatosis^25^.

Other studies have found that some microbial species can reduce the risk of formation of atherosclerotic plaque. For example, research in both patients and mouse models has demonstrated that relative reduction in butyrate‐producing *Roseburia* and *Eubacterium* bacteria was inversely associated with development of atherosclerotic lesions^20^, ^26^. Moreover, recent studies in both mice and humans have identified that treatment with agents used in atherosclerotic disease, such as bicyclol and berberine, affects atherosclerosis‐related indicators by changing the gut microbiota^27^, ^28^. Gut‐related bacteria in atherosclerotic plaque were found to correlate with the clinical parameters of alanine aminotransferase, total cholesterol, and fibrinogen levels, and these gut microbes may influence inflammation status in the host^29^.

These findings indicate that the gut microbiota plays a mediating role in cardiovascular disease. Therefore, changing the composition of the gut microbiota could be expected to be an effective prophylactic and therapeutic strategy.

### The gut‐oral microbiome crosstalk in atherosclerosis

In addition to the gut microbiome, the oral cavity is also a source of microbes that may be involved in cardiovascular disease. Several types of oral bacteria, including *Porphyromonas gingivalis* and *Treponema denticola*, have been identified in atherosclerotic plaques^30^. Several studies have shown associations between periodontal disease and cardiovascular disease and between poor oral hygiene and acute myocardial infarction^11^, ^31^, ^32^. Furthermore, oral or intravenous administration of bacteria in the oral cavity was found to aggravate atherosclerosis in a mouse model^33^, ^34^, ^35^. Studies have demonstrated that microbial species can be transmitted from the oral cavity to the gut and that the oral and gut microbiota can interact with each other^11^. Given the association between dental health and both endothelial dysfunction and atherosclerotic disease, the gut may serve as a niche or entry path for hematogenous spread of bacteria that are pathogenic in the oral cavity^36^, ^37^. These studies suggest that bacteria in the oral cavity are associated with cardiovascular disease and may spread to the circulation to affect the growth and stability of atherosclerotic plaques.

However, the microbial species that play a dominant role in cardiovascular disease remain unclear, and the mechanisms by which the microbiome affects development of atherosclerosis should be a focus in future research.

## GUT MICROBE METABOLITES

### SCFAs

The human gut cannot digest complex carbohydrates in the form of dietary fiber, but gut microbes can convert dietary fiber into SCFAs by fermentation^38^. SCFAs are saturated fatty acids, usually containing one to six carbon chains. The most common SCFAs found in the gut are acetic acid, propionic acid, and butyric acid^39^.

The abundance of conditional pathogenic microbes has been reported to be higher and that of butyrate‐producing bacteria to be lower in patients with atherosclerosis^26^. Furthermore, colonization by proinflammatory gut microbes that accelerate atherosclerosis was found to be related to a lower abundance of SCFA‐producing bacteria in feces and decreased levels of SCFAs in the cecum^23^.

Butyrate supplementation in *ApoE* ^−/−^ mice on a high‐fat diet promoted efflux of cholesterol via macrophages by promoting expression of ABCA1, thereby inhibiting atherosclerosis and hepatic steatosis and regulating the intestinal microbiota^40^. Administration of *Roseburia intestinalis*, a colonizing butyrate‐producing bacterium, reduced inflammatory markers and improved atherosclerosis in mice receiving dietary plant polysaccharides^26^. This finding suggest that changes in diet and microbiota interactions could affect cardiovascular disease and that interventions by increasing butyrate‐producing bacteria may prevent atherosclerosis.

Another study in *ApoE* ^−/−^ mice by Haghikia et al. revealed that administration of propionate attenuated the arteriosclerotic phenotype and the hypercholesterolemia induced by a high‐fat diet via a route that was independent of the gut microbiota^41^. This study also found that propionate increased the levels of intestinal regulatory T cells and interleukin‐10, and inhibited the expression of Npc1l1, an important cholesterol transporter in intestinal epithelial cells, thereby decreasing the serum cholesterol level. Furthermore, Bartolomaeus et al. found that propionate protected against cardiac hypertrophy, fibrosis, vascular dysfunction, and hypertension^42^.

### Trimethylamine N‐oxide

TMAO is synthesized in the host liver and derived primarily from bacterial metabolism of choline and phosphatidylcholine in the diet^43^. The gut microbiota plays an essential role in the production of trimethylamine (TMA), a precursor of TMAO. Some of the bacteria in the gut produce TMA‐lyase, either directly from dietary intake or indirectly from production. TMA‐lyase converts choline, betaine, carnitines into TMA, which enters the portal circulation in the liver and is oxidized by flavin‐containing monooxygenase (FMO) to produce TMAO^44^. TMAO is considered to an independent risk factor for cardiovascular disease, and elevated levels of TMAO have been associated with an increased risk of heart failure, heart attacks, and peripheral artery disease^45^, ^46^.

An analysis of data from more than 3000 patients with atherosclerosis identified a significant correlation between disease phenotype and plasma levels of intestinal microbiota metabolites, particularly TMAO and p‐cresyl sulfate^47^. Studies of the gut microbiota have shown that the bacterial enzyme choline TMA‐lyase converts choline into TMA, which is then converted into TMAO via flavin‐containing monooxygenase 3 (FMO3) in the host liver. Jiang et al. recently identified intestinal bacteria encoding FMOs in the gut and that TMAO was produced through a choline–TMA–TMAO pathway^48^. They also found that *Lachnoclostridium saccharolyticum* and choline in *ApoE* ^−/−^ mice could increase serum TMAO levels and aggravate atherosclerosis. This finding indicated that *L. saccharolyticum* could convert choline into TMA, thereby promoting the onset and progression of atherosclerosis. Intake of l‐carnitine, which is an important dietary precursor of TMA, has been reported to increase the risk of atherosclerosis via the gut microbiota^49^. Recent studies of gut microbiota have found that *Firmicutes* contains *bbu* genes, which could convert the intermediate γ‐butyrobetaine into TMA in the anaerobic environment of the gut^50^, ^51^. This finding may further facilitate development of targeted dietary intervention strategies to limit formation of TMA from l‐carnitine.

Studies in mice and humans have shown that raw garlic and allicin (the main compound in raw garlic) decrease the production of TMAO by regulating the gut microbiota, thereby decreasing the risk of cardiovascular disease^52^. Chen et al. found that resveratrol inhibited the production of TMA by reshaping the gut microbiome, reducing the TMAO level, and protecting against the onset of atherosclerosis^53^. By systematic analysis of TMA‐producing bacteria in the human gut microbiota, Li et al. found that TMA‐lyase‐containing *L. saccharolyticum* WM1 was abundant in patients with atherosclerosis and could convert choline into TMA^48^.

Oral berberine may inhibit the activity of bacterial choline TMA‐lyase and FMO and reduce the production of bacterial TMAO through a mechanism like that of vitamins, thereby improving atherosclerosis in animal models and patients^28^. The study revealed a novel gut microbiota‐related mechanism by which berberine improves cardiovascular health, suggesting that berberine is a potential treatment for atherosclerosis.

Recently, Chen et al. demonstrated that targeted loss of proline‐rich/serine coiled‐coil protein 1 (PSRC1) could affect the intestinal flora and liver FMO3, accelerate the production of TMAO, and promote atherosclerosis^54^, suggesting that PSRC1 could potentially regulate the intestinal microbiota and alleviate atherosclerosis.

Overall, the evidence suggests that TMAO or TMAO‐producing bacteria could affect the onset and progression of atherosclerosis. Therefore, targeting TMAO‐producing bacteria may be a potential therapeutic approach for atherosclerosis.

### Bile acids

In addition to a chronic inflammatory response and abnormal metabolism of cholesterol, the onset and progression of atherosclerosis are also associated with the metabolism of bile acids^55^. Charach et al. demonstrated that the ability to excrete bile acids was significantly lower in these patients with coronary heart disease than in healthy controls^56^. They also found that the total bile acid and deoxycholic acid levels were significantly lower in these patients, as was the stone cholic acid level in stool. These observations indicate that an inability to excrete bile acids effectively may also contribute to development of coronary atherosclerosis.

Bile acids are an important signaling factor and regulate multiple biological processes, including the metabolism of lipids, glucose, energy, inflammation, and the immune response through the bile acid receptor (FXR) and G protein‐coupled receptor 5 (TGR5)^57^. FXR is mainly expressed in the liver and intestines and is involved in the regulation of lipid metabolism, especially the transport, synthesis, and use of triacylglycerol^58^. Bile acids reduce the incidence of atherosclerosis by activating FXR. Hanniman et al. found that *FXR* ^−/−^ *ApoE* ^−/− ^double knockout mice developed more severe atherosclerosis after being fed a high‐fat and high‐cholesterol diet^59^. The favorable effect of FXR on the formation of atherosclerotic plaque was partly attributed to improve abnormal lipid metabolism. Bile acids also reduce the development of atherosclerosis via activation of TGR5^60^. Miyazaki et al. showed that TGR5 has an anti‐atherosclerosis effect^61^. Bile acids that bind taurine and glycine could reduce the nuclear factor kappa‐B level by activating TGR5, leading to a decrease in proinflammatory cytokine levels, further inhibiting inflammation in atherosclerotic plaques and development of atherosclerosis^60^. Other researchers have shown that deficiency of both FXR and TGR5 exacerbates development of atherosclerosis^61^. Activation of FXR and TGR5 may be an important strategy for the prevention and treatment of atherosclerosis.

*ApoE* ^−/−^ mice fed on a high‐cholesterol diet showed an increase in the serum FGF19/FGF15 level that was regulated by FXR in the intestines and positively correlated with the circulating ceramide level, inhibiting the intestinal FXR/SMPD3 axis^62^. Serum ceramide levels decreased and atherosclerosis improved after supplementation with glycoursodeoxycholic acid or GW4869.

Bile salt hydrolases play a critical role in bile acid metabolism and are present in a variety of intestinal bacteria^63^. Chen et al. found increased abundances of *Lactobacillus* and *Bifidobacterium* in *ApoE* ^−/−^ mice after treatment with resveratrol, which improved the activity of bile salt hydrolyases and enhanced the synthesis and excretion of bile acids^53^.

### Amino acids

Gut microbiota can also produce many other metabolites, for example, aromatic amino acids (e.g., phenylalanine, tryptophan, and tyrosine). There is evidence to suggest that patients with advanced atherosclerosis have significantly low plasma levels of tryptophan, which is a metabolite produced by microorganisms^64^. Ling et al. found that the bacterial metabolite indole‐3 propionic acid, a metabolite from tryptophan, promoted reverse transport of cholesterol in macrophages via the miR‐142‐5 p/ABCA1 axis^65^. Amino acids may become an intervention target in atherosclerotic cardiovascular disease.

## TREATMENT OF ATHEROSCLEROSIS

**Figure 2:**
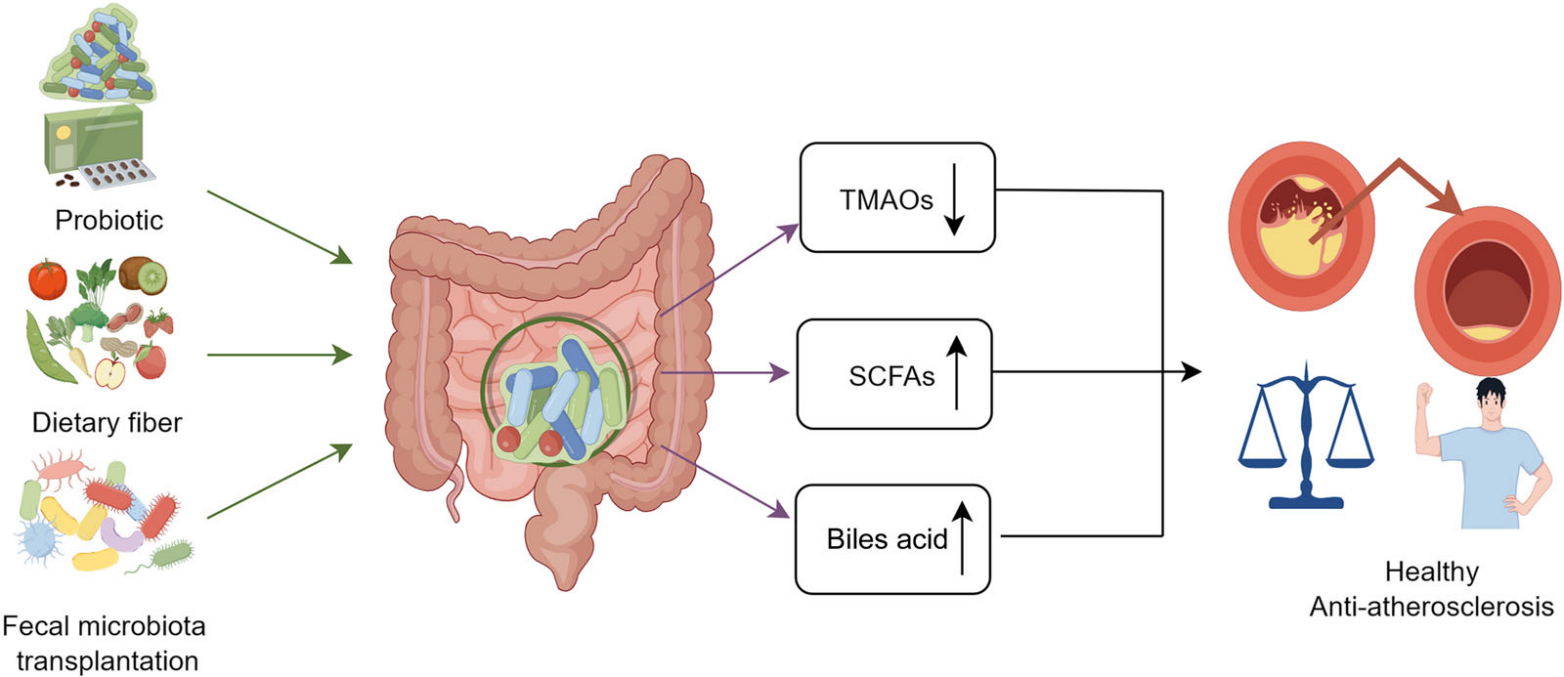
Manipulating the structure of the gut microbiota to ameliorate atherosclerosis. Therapeutic strategies (i.e., supplementation with probiotics and prebiotics or fecal microbiota transplantation) for treating atherosclerosis are mainly dependent on reshaping the structure, composition of gut microbiota, and finally regulating metabolites produced by gut microbiota to promote anti‐atherosclerosis process. This figure was drawn by Figdraw (ID:SPSUI88d68).

Increasing evidence indicates that gut microbes are strongly associated with the onset and progression of atherosclerosis. Therefore, therapeutic strategies for treating atherosclerosis based on the gut microbiota could be a novel approach. Manipulation of the structure and composition of the gut microbiota by supplementation with probiotics and prebiotics or fecal microbiome transplantation could potentially alleviate atherosclerosis (Figure 2).

### Probiotics

Although harmful bacteria promote atherosclerosis, there are also substantial amounts of beneficial bacteria in the gut that could have both preventive and therapeutic effects on the onset and progression of atherosclerosis. Chan et al. found that supplementation with *Bifidobacterium longum, B. breve, B. infantis, Lactobacillus acidophilus, L. paracasei, L. plantarum, Streptococcus thermophilus*, and *L. bulgaricus* significantly reduced the size of atherosclerotic plaques in the arteries of *ApoE* ^−/−^ mice and had favorable effects with reducing pro‐inflammatory adhesion molecules and risk factors that induce plaque rupture^66^.

Zhang et al. found that conventional treatment had a more marked effect when used in combination with Probio‐M8, which contains several probiotics, than when it was administered with a placebo in patients with coronary artery disease^67^. They suggested that the beneficial effect may be achieved by increasing the abundance of beneficial bacteria species, reducing serum TMAO/TMA levels, regulating the level of metabolites, including specific amino acids, and affecting the gut–spindle and gut–brain axes. Their study showed that probiotics in combination with traditional therapies could effectively reduce the symptoms of visceral/heart‐related diseases, including coronary artery disease.

Qiu et al. reported that *L. plantarum* ZDY04 significantly reduced the serum TMAO level and the cecal TMA level by modifying the composition of the gut microbiome and demonstrated that it may be an alternative strategy for amelioration of TMAO‐induced atherosclerosis^68^. *Akkermania muciniphila* is an intestinal probiotic that can effectively improve glucose and lipid metabolism in the host. Li et al. found that *A. muciniphila* prevented and controlled inflammation in atherosclerotic lesions by reducing infiltration of macrophages, levels of proinflammatory cytokines, levels of chemokines, and endotoxemia^69^.

### Prebiotics

#### Dietary fiber

A recent study in a population of Southeast Asian descent found that a healthy plant‐based diet was associated with reduced cardiometabolic risk, while unhealthy plant‐based dietary items (e.g., refined grains, pickles, fried snack foods, coconut, potatoes, desserts, and sugar‐sweetened beverages) were not^70^.

Hutchison et al. identified that attenuation of atherosclerosis by dietary fiber was mediated by the gut microbiome^13^. Sakurai et al. also showed that dietary alpha‐cyclodextrin, a cyclic polymer of glucose, decreased atherosclerosis by modifying the composition of cecal bacteria^71^. Other studies have similarly demonstrated that high intakes of dietary fiber, such as dietary inulin and pectin, can attenuate atherosclerosis^72^, ^73^, ^74^. These findings suggest that dietary fiber could halt the onset and progression of atherosclerosis.

#### Polysaccharides

Studies have shown that zinc levels are markedly lower in individuals who are obese than in healthy nonobese individuals and that low zinc status is associated with obesity‐related metabolic disorders^75^. Zinc supplementation may be a potential strategy to combat obesity. Polysaccharides/oligosaccharides contain many carboxyl and hydroxyl groups and are excellent ligands for metal ions, including zinc ions^76^. Polysaccharides/oligosaccharides are also important prebiotics and widely used. Li et al. recently found that a novel zinc supplement based on Ulva oligosaccharides could improve the structure of the intestinal flora and attenuate dyslipidemia while reducing body weight in obese mice, suggesting the potential of supplementation to improve the health of obese individuals^77^.

Hoving et al. found that mannose oligosaccharides could prevent worsening of atherosclerosis by reducing serum cholesterol levels and that these oligosaccharides may increase the butyric acid level in the cecum and promote excretion of bile acids by influencing the gut microbiome^78^. Li et al. demonstrated that polysaccharides from *Laminaria japonica* suppressed atherosclerosis by increasing activity in the autophagy pathway^79^. Red algal polysaccharide has been investigated and may be a potential treatment for atherosclerosis^80^. In summary, these studies provide innovative clues for development of further strategies for prevention and treatment of atherosclerosis.

### Small molecule compounds

The current methods for regulating the gut microbiome can be divided into three categories, namely, probiotics, prebiotics, and small molecules, among which probiotics and prebiotics are widely used. However, the safety of probiotics is unclear and their colonization rates are unstable, and although prebiotics can improve the composition of the flora, the direction of the composition cannot be predicted or controlled precisely. Researchers at the Scripps Research Institute have developed a screening method for identifying molecules that can selectively affect the growth of bacteria, and identified two types of annular molecules, namely, d and L‐α‐peptides, that can reverse a high‐fat diet of gut microbes into a low‐fat diet group. In mice with atherosclerosis induced by a high‐fat diet, 2 weeks of treatment with d,l‐α‐peptide molecules reduced cholesterol levels by 36% and 10 weeks of treatment reduced the area of atherosclerotic plaque by about 40%^81^. Previous research has shown that some cyclic peptides with an even number of d,l‐α‐amino acids (usually six or eight) could be selectively embedded in bacterial cell membranes, affecting the transmembrane potential and ion concentration gradient, disrupting the transmembrane transport of substances, and finally preventing bacterial growth or even depleting bacteria^81^.

Wang et al. showed that 3,3‐dimethyl‐1‐butanol, a structural analog of choline, attenuated the development of atherosclerosis by inhibiting the activity of microbial TMA‐lyases and the production of TMA and TMAO^82^. Liu et al. also identified a *Ganoderma* meroterpene derivative that enriched the abundance of *Parabacteroides merdae*, enhanced catabolism of branched‐chain amino acids, and improved obesity‐associated atherosclerosis^83^. Overall, these results have revealed a novel mechanism of gut microbiota‐mediated progression of atherosclerosis and provided potential novel intervention strategies for regulation of the microbiota to improve cardiovascular health.

### Perspectives

Atherosclerosis is a complex disease with underlying mechanisms that are still not well understood. Numerous studies have shown that the gut microbiota plays a pivotal role in the onset and progression of atherosclerosis. A healthy gut microbiota can protect against cardiovascular disease, while a dysfunctional gut microbiota may increase the risk of developing the disease. This review provides an overview of many studies that have described the possible relationship between the gut microbiota and atherosclerosis. The gut microbiota can alter cholesterol metabolism, influence the immune system, produce bacterial metabolites (e.g., SCFAs), and translocate into plaque tissue to influence atherosclerosis.

With advances in the technology used in research on the gut microbiota, its importance in terms of health is increasingly being recognized. TMAO is a typical cardiovascular‐related metabolite and its production requires bacteria, indicating that the gut microbiota plays a key role in the onset and progression of atherosclerosis. Targeted regulation of the gut microbiome provides novel strategies for the treatment of atherosclerosis in clinical practice.

Reshaping the gut microbiota by fecal transplantation or by probiotic and dietary interventions has been shown to be safe and effective for the management of atherosclerosis and its associated risk factors. These therapies have shown promising results in animal and human studies. However, their precision and rational use in humans and their safety await confirmation in larger‐scale clinical trials. There is no single strain within the gut microbiota that plays an absolute role, and the relationships between the different species of bacteria and the host are key. Future studies should focus on gut microbiota‐related metabolites and their mechanisms, the interaction between “good” and “bad” bacteria, and how to tailor modifications of the microbiome to the individual.

Multiple studies have shown that oral bacteria are closely associated with atherosclerosis. The oral and intestinal flora are not completely independent systems. In intestinal endothelial dysfunction, oral bacteria may enter the bloodstream and affect the onset of atherosclerosis by interfering with host metabolism and impacting the level of the inflammatory response. Therefore, changing oral health status may be another novel strategy for the treatment of atherosclerosis and have economic benefits at the population level.
